# Physical Training Considerations for Futsal Players According to Strength and Conditioning Coaches: A Qualitative Study

**DOI:** 10.3390/sports13040126

**Published:** 2025-04-18

**Authors:** Rafael Albalad-Aiguabella, David Navarrete-Villanueva, Elena Mainer-Pardos, Oscar Villanueva-Guerrero, Borja Muniz-Pardos, Germán Vicente-Rodríguez

**Affiliations:** 1 Health Sciences Faculty, Universidad San Jorge, Autov A23 km 299, Villanueva de Gállego, 50830 Zaragoza, Spain; ralbalad@usj.es (R.A.-A.); epardos@usj.es (E.M.-P.); oscarvillanuevaguerrero@gmail.com (O.V.-G.); 2 EXER-GENUD (Growth, Exercise, NUtrition and Development) Research Group (S72_23R), FIMS Collaborating Center of Sports Medicine, University of Zaragoza, 50013 Zaragoza, Spain; dnavarrete@unizar.es (D.N.-V.); gervicen@unizar.es (G.V.-R.); 3 AgroFood Institute of Aragon (IA2), Universidad de Zaragoza, 50009 Zaragoza, Spain; 4 Faculty of Health Science, University of Zaragoza, Domingo Miral, s/n, 50009 Zaragoza, Spain; 5 Physiopathology of Obesity and Nutrition Networking Biomedical Research Centre (CIBERObn), Instituto de Salud Carlos III, 28029 Madrid, Spain; 6 Exercise and Health Spanish Research Network, 50009 Zaragoza, Spain; 7 Faculty of Health and Sport Science (FCSD), University of Zaragoza, Ronda Misericordia 5, 22001 Huesca, Spain; 8 Department of Physiatry and Nursing, University of Zaragoza, 50009 Zaragoza, Spain

**Keywords:** sport, futsal, performance, coaching, player monitoring, individualization

## Abstract

The professionalization of futsal requires greater physical demands on players, requiring strength and conditioning coaches to manage loads, optimize performance, and prevent injuries. This study aimed to describe the current practices of high-level strength and conditioning coaches and determine the elements needed to optimize their performance. Two video-recorded focus groups consisting of eight strength and conditioning coaches from the Spanish futsal league’s first and second divisions were transcribed, translated, and analyzed using a content analysis approach with open-ended questions on physical preparation and current practices. Results showed that strength and conditioning coaches prioritized five main areas: (1) competitive demands, (2) training load control and monitoring, (3) injury risk mitigation strategies, (4) contextual factors and interpersonal relationships, and (5) training methodologies to optimize performance. However, they also claim to deal with several limitations such as lack of time, limited resources and access to facilities, insufficient staff, problems related to combining sport with other activities (e.g., work), or the difficulty to individualize, which limits the optimization of their practices. Based on these findings, practical applications include implementing neuromuscular and strength training sessions at least twice a week, using cost-effective load monitoring tools (e.g., RPE and wellness questionnaires) to manage workloads, individualizing training programs to address the specific demands and characteristics of each player, and fostering close multidisciplinary collaboration to optimize performance and reduce injury risks. These insights can guide current and aspiring strength and conditioning coaches toward optimized practices. This study can assist novice strength and conditioning coaches in identifying the key focus areas of elite physical trainers and understanding their challenges and limitations, fostering collaboration among sports professionals to create a more optimized environment.

## 1. Introduction

Futsal is a team sport of cooperation and opposition and it is considered an intermittent discipline with incomplete recovery periods, where high-intensity efforts predominate and occur continuously throughout the match [1,2,3]. Competitive schedules are often saturated with numerous competitions, with little time for recovery and training between matches [4]. In recent years, the physical demands of futsal have increased, leading to an ever-growing demand for players’ technical, tactical, physical, physiological, and psychological capabilities [5,6]. This demand has required strength and conditioning coaches (S&CCs) to optimize training methodologies to maximize athletic performance, prepare players for competition, and maintain high physical conditioning throughout the season [7,8].

The role of an S&CC is complex and heterogeneous, encompassing the development and maintenance of diverse and essential physical capacities for optimal performance [9]. S&CCs are focused on planning adequate workloads, balancing training and recovery to ensure optimal periodization while minimizing injury risk [10,11,12,13], and are also required to collaborate within multidisciplinary teams [14]. Different tools for load quantification and monitoring are frequently used in both training and competitions, providing valuable data that help in the decision-making process during training [15,16]. The training load can be understood according to two main components: internal and external loads [17]. Internal load refers to physiological and psychological responses to training [18], with commonly used markers such as heart rate [19,20], biochemical variables [21,22], subjective perception of effort (RPE), and well-being questionnaires [23,24]. External load refers to the objective and measurable demands of training [25], with the emergence of tracking technologies, such as global positioning systems (GPS), local positioning systems (LPS), or indoor accelerometers [26,27], aiding in the assessment of key variables, such as total distance covered, player load, accelerations, deceleration, or distance covered at high intensity, among others [1,7]. Strength and conditioning coaches must enhance a range of physical capacities in players, including strength, sprint performance, and change-of-direction ability. In addition to routine futsal training, several studies have demonstrated that incorporating supplemental training, such as resistance training, plyometrics, agility exercises, or combined strength–speed workouts, can significantly improve these capacities [28,29,30,31]. Evidence suggests that one to two additional training sessions per week, effective from low to moderate intensities and lasting between 15 and 45 min, depending on the type of training, are sufficient to elicit meaningful performance improvements [15,29,31].

Additionally, individualizing training loads according to individual player characteristics such as age, gender, training history, or physical fitness is also essential for S&CCs at the professional level [32,33,34]. Notably, optimizing the physical preparation requires accounting for other confounding variables, such as competition schedules, club environments, player characteristics, and interpersonal relationships [35,36]. For this purpose, S&CCs should have a wide perspective, so all these factors are considered.

While an increasing number of studies have analyzed the physical demands of futsal [7,8,35,37,38], little research has been conducted on the usual practices of S&CCs and their influence on the aforementioned variables involved in their practice. In other sports, previous studies have explored the methodologies used by S&CCs through closed-ended surveys and questionnaires [39,40,41,42,43,44,45]. To date, in futsal, only one study has been found that analyzed the practices of S&CCs [15]. However, this research employed closed-ended survey questions, which limited the depth of the insights.

Given this gap in the literature and the inherent strengths of the focus group with open questions, the present study conducted focus groups with futsal S&CCs to achieve two main objectives: (1) to identify the fundamental principles and specific practices guiding their training methodologies and (2) to determine the nonconditioning-related factors that significantly impact their professional responsibilities (e.g., related to context, inter-individual relationships). The findings of the present work contribute to both research and applied practice, providing valuable insights for futsal S&CCs.

## 2. Materials and Methods

### 2.1. Study Design

This study used a descriptive, qualitative, cross-sectional design to analyze and understand the usual practices of futsal S&CCs (first or second-division) during the competitive season to increase the physical performance of their players and prevent injuries. Two focus groups were performed in October 2023. Each group comprised four S&CCs from the first- or second-division men’s and women’s futsal teams of the Spanish league. Access to data was exclusively limited to the researchers. Data were encoded to ensure anonymity and were destroyed once the study was completed.

### 2.2. Participants

Initially, 10 professional S&CCs from the first- and second-division men’s and women’s futsal teams in the Spanish league were contacted to voluntarily participate in the study. Eight of the 10 S&CCs responded positively and agreed to participate in this research. Before conducting the focus group discussions, all participants signed an informed consent to participate in the research and agreed to have the focus group video recorded. The age of the S&CC participants was 31.1 ± 4.82 years, with a wide experience in sports performance (8.1 ± 4.78 years of experience) and, in particular, in futsal performance (4.8 ± 3.31 years of experience). Table 1 presents the participants’ characteristics.

Participation in the focus group was voluntary. If, at any time during the focus group, any participant considered that any of the topics being discussed were inappropriate or put them in an uncomfortable situation, they were free to leave the discussion or group without providing any justification.

For participants to be included in the study, they were required to meet the following inclusion criteria: (1) actively working during the data collection period; (2) employed by teams competing in the first or second division; (3) eligible regardless of team gender; and (4) possess at least a Bachelor’s degree in sport sciences. The Clinical Research Ethics Committee of Aragon (CEICA) approved the research project. Ethics was initially obtained for this study in November 2023 (PI23-456), but because this intervention was integrated into a research project that included other experimental trials, an addendum was made to our initial ethics application in April 2024 (PI23-456). The research project was conducted in accordance with the ethical standards of the 1975 Declaration of Helsinki (revised at the 64th General Assembly, Fortaleza, Brazil, October 2013). Confidentiality was guaranteed following legal regulations on data confidentiality established by Organic Law 15/1999 of 13 December, Protection of Personal Data, and Organic Law 3/2018 of 5 December, Protection of Personal Data and Guarantee of Digital Rights.

### 2.3. Procedures

**Figure 1:**
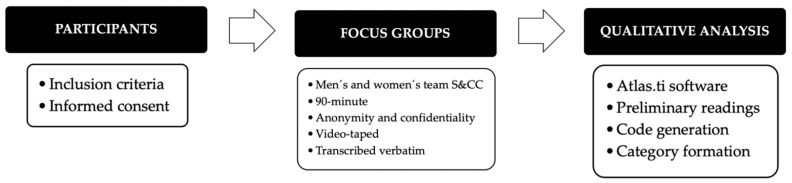
Data collection process.

Once the participants who met the inclusion criteria were selected and their consent was obtained, each participant was randomly assigned to one of the two focus groups. Both groups were required to include S&CCs from both the men’s and women’s teams. The focus groups began by explaining the research’s aim, the topics to be discussed, and the estimated duration. In addition, participants were informed of the anonymity and confidentiality of their information, opinions, comments, and use. Each S&CC participated in a 90-min virtual focus group composed of four S&CCs conducted through Microsoft Teams (v. 25044.2407.3474.4587, Microsoft Corporation, Redmond, WA, USA) and led by the investigator. The structure was similar in both focus groups, and S&CCs could provide additional information during the dynamics. The focus groups were videotaped and transcribed verbatim. Participants did not receive any financial or other compensation for their participation in the focus group. Figure 1 shows the data collection process.

### 2.4. Qualitative Analysis

A combined thematic and structural analysis of the focus group data was conducted to examine not only what was said but also how it was communicated by the participants [46]. The thematic analysis focused on capturing the meaning, description, and interpretation of the data’s content, while the structural analysis explored the connotative aspects of language, including the use of specific expressions and linguistic forms [46]. Before starting the analysis, all data were prepared by verbatim transcription, anonymization, and aggregation of the information obtained using the aforementioned techniques.

**Figure 2:**
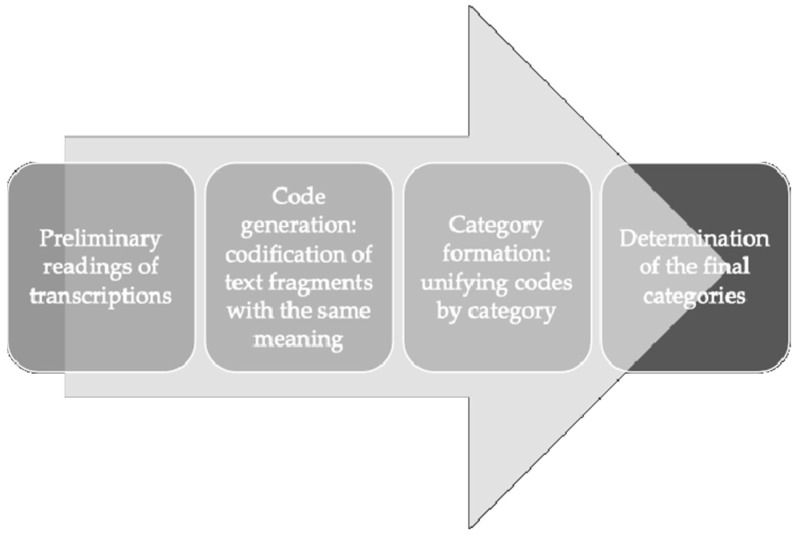
Qualitative analysis process.

For the qualitative analysis of the focus group’s content, the texts were imported into the Atlas.ti qualitative analysis software (v.23.4.0, Berlin, Germany) to facilitate systematic coding and categorization. The analysis was performed by two independent researchers (RAA and OVG), who conducted three thorough preliminary transcript readings. During the subsequent coding phase, recurring quotes were identified, and keywords emerging from the text were used to create codes. These codes were then grouped into categories that encompassed similar thematic content, ensuring a systematic and comprehensive understanding of the focus group discussions [46]. Figure 2 illustrates the qualitative analysis process.

### 2.5. Validity and Robustness

To ensure data quality, diverse triangulation techniques were used. In this study, triangulation involved a comprehensive search of the scientific literature and focus group interviews. The interview questions were developed based on the literature review, and data from the second focus group were triangulated with those from the first. Moreover, different members of the research team (RAA and OVG) independently conducted triangulation to compare and contrast the information and enhance credibility. This multifaceted approach enabled us to identify convergences and divergences in our data, thereby strengthening the validity of our findings. The reliability of the results was verified by including verbatim statements from the participants, and the entire work process was supervised by different team members who were not directly involved in the analysis of the results. In addition, the study’s rigor was ensured by following the COREQ EQUATOR guidelines and applying the reliability criteria for qualitative research following Lincoln and Guba [47,48].

## 3. Results

The thoughtful analysis of the focus groups led to the following five general categories: (1) Analysis of the futsal performance model and competitive demands; (2) Load monitoring; (3) Injuries; (4) Contextual and relational factors; (5) Training methods and limitations. Table 2 presents a summary of the main results for each category identified through qualitative analysis.

### 3.1. Analysis of Futsal Performance Model and Competitive Demands

#### 3.1.1. Futsal Performance Model

Regarding the physical capacities that determine futsal performance, there was a consensus on the dominance of strength training, mainly of the lower body, and this was agreed to be used for both sports performance improvement and injury prevention. Aerobic training is also commonly performed through intermittent and repetitive high-intensity actions, prioritizing both aerobic and anaerobic energy systems. These actions occur continuously during competition and are characterized by brief, active, and incomplete recoveries. In addition, speed training, where acceleration plays a fundamental role, is crucial for player preparation. No sex differences (male and female) were found between the S&CC practices in this section.

“Based on the current trends in futsal, training programs should consider strength training as a key element.” I consider this one of the main pillars of training plans together with the aerobic-anaerobic training”

(PT 1)

“In addition to the previously mentioned strength and endurance training, (…) players should also be able to cover very short distances as quickly as possible, making acceleration a key component of their performance.”

(PT 5)

“(…) and let’s not forget it is essential to incorporate the ability to repeatedly perform high-intensity actions, such as accelerations, decelerations, fights, or offensive and defensive transitions, into training programs, as these are essential for player’s physical conditioning”

(PT 3)

In addition, one participant added that for the optimal development of all these capabilities and the prevention of injuries, we should not fail to include joint mobility and range of motion exercises in our training programs.

“To complement the previously mentioned aspects, (…) I would emphasise that joint mobility and range of motion training should always be integrated into our training programs”

(PT 4)

#### 3.1.2. Competitive Demands

To identify whether S&CCs considered the competitive demands of program exercise training, we found that most of them prioritized playtime and player position, distance covered and specific actions. Additionally, these variables were mainly used to control the weekly individual and team workloads. It should be noted that only two of the participants had worked in their teams with advanced GPS technology, who highlighted that this technology enabled them to have a more real and precise quantification of numerous external load variables (accelerations, decelerations, distances covered, etc.). This method was claimed to help adjust training exercises and weekly loads more reliably based on the obtained data. There were no differences in S&CC practices according to sex. However, male teams generally have greater economic resources and a higher prevalence of GPS usage, which optimizes their training load monitoring.

“Yes, I reckon the value of assessing competitive demands, but ‘playing time’ is the only variable I include, given my limited access to GPS technologies”

(PT 1)

“For instance, if one player participated 25 min and another 12 min in a match, an alternative strategy would be to individualize the first weekly training session to compensate the workload differences”

(PT 5)

“Each playing position has different physical demands in terms of strength, distance covered, and fighting actions. For instance, pivots engage in challenging actions to gain and maintain positions. Therefore, in addition to general training, these players require a greater emphasis on strength development”

(PT 6)

“I primarily focus on player profiles, which indirectly influence the training plan in terms of running distances. For instance, if I have a player whose role is pivot, I avoid programming excessive distances in a fixed pivot system. Therefore, I ensure that the prescribed distances closely align with the specific competitive demands. Additionally, in discussions with the coach, he specifically requests detailed insights into certain player`s conditioning levels”

(PT 2)

“Using GPS technology provides us with a distinct advantage in understanding the actual competitive demands”

(PT 3)

### 3.2. Load Monitoring

In the second block, participants shared their views on load control, its importance, and the tools they used to manage it. All participants, regardless of the sex of the team, agreed that having absolute control over training loads through proper periodization is crucial. Although training planning should always follow a holistic approach, it should be primarily focused on the short term to meet the immediate demands of competition.

“It is essential to meticulously monitor loads and plan them at weekly or microcycle level, given the numerous demands of the season schedule”

(PT 6)

Another aspect highlighted by the S&CCs, for both men’s and women’s teams, is the relevance of implementing different daily questionnaires (wellness, subjective perception of effort, muscular quality, quality of recovery, etc.) to check the players’ physical condition and thus be able to adapt the training sessions. S&CCs working with women’s teams reported that female athletes tend to be less accustomed to using these questionnaires compared to their male counterparts, thereby requiring additional time to familiarize themselves with the process.

“The questionnaires provide me with a wealth of information about how the players are performing” (PT 5), and “based on the questionnaires, you can adjust the loads and modify the type of session”

(PT 8)

New technologies, which are more prevalent in men’s teams due to greater financial support, enable S&CCs to more precisely control training and match loads, allowing for adjustments not only in the load itself but also in recovery protocols and training exercises.

“Now that I have GPS technology, I have access to plenty of information. Ultimately, this approach enables us to precisely identify competitive demands, resulting in significant improvements in load control and better structuring loads throughout the microcycle. For instance, you can verify whether you require more acceleration and deceleration on Tuesdays and whether you may need a more maximum-intensity distances on Wednesdays. Consequently, further adjustments can be made.”

(PT 4)

### 3.3. Injuries

This third category of results gathers the participants’ perspectives on various topics of futsal injuries and the strategies employed by S&CCs to minimize injury risk.

#### 3.3.1. Main Injury

Regarding the main injuries typically observed in male and female players, S&CCs predominantly highlight ligament and muscle injuries, most commonly located in the ankle, knee, hip, quadriceps, and adductors.

“In my experience, I have encountered more ankle sprains than any other injury. Additionally, I have dealt with tendinopathies in various areas, most commonly in psoas and in the knee through patellar problems, and adductor overload”

(PT 1)

“Then in the knee, there have been lateral or medial sprains” (PT 6), and “regarding the quadriceps, I have mainly witnessed overload injuries, although occasionally there has been a tear”

(PT 4)

“Now I am concerned because I already have another case of pubalgia, an injury I experienced last year” (PT 7), “I have also encountered a case of pubalgia, a long and challenging injury to manage”

(PT 6)

“I would also note pain or discomfort in the lower back”

(PT 6)

In this regard, some S&CC participants noted that some injuries could be linked to the type of court on which training sessions are conducted or to the athlete’s gender.

“The surface of the court is also a key factor in determining the type of injuries that can occur during the season. Last year we played on a very hard court, and three players suffered bone edema due to hard impacts against the court during different actions during the game. I had never seen this injury before, and it caused long-term absences of the players. In addition, some players suffered Achilles tendon discomfort due to the hardness of the court, especially when exposed to low temperatures during winter.”

(PT 4)

“Girls are more exposed to a greater extent than men to suffer anterior cruciate ligament injuries due to their physical and anatomical characteristics.”

(PT 2)

“In females, I have found a higher prevalence of discomfort and injuries in the adductor muscles.” Anterior cruciate ligament injuries are also frequently observed, which is a very frequent injury in female players nowadays”

(PT 3)

However, concerning the S&CCs’ perception of injury incidence in futsal, they believe that the overall injury rate is low, regardless of sex. Most of the time, these injuries are minor and can be managed throughout the week, allowing the players to participate in the competition.

“Given the current level of demand in futsal, I believe the injury rate is not excessive, especially considering the limited resources available, the long trips (generally in bus), the highly-demanding season schedule…”

(PT 4)

#### 3.3.2. Minimization of Injury Risk

The participants emphasized that the role of S&CCs is not to skip injuries fully, which is inherent in high-level sports, but to try to minimize injury risk through the various tools available and diverse strategies that can be implemented. They noted that these strategies extend beyond merely performing low-intensity exercises, which are commonly known as “preventive”. Furthermore, some variables cannot be controlled and depend directly on the players and cannot be fully controlled by the S&CCs; thus, the responsibility for injuries is shared between the players, S&CCs, and the rest of the technical team.

“It is widely accepted in the futsal community that muscle injuries are physical trainers’ fault, but this is not as simple; it is a responsibility shared equally between the physiotherapist, coach, player, and physical trainer. The physical trainer could potentially program a meticulous training plan with individualized loads, etc., but if the coach is not on the same page and works individually with his own training plan, it is impossible to build synergy and teamwork. I have worked with coaches who prepared down to the smallest detail: Which day do we train what? Which court size should we use, depending on the number of players? What time? However, I have worked with other coaches who say, “No, this is my way of training; we are going to do this, and that is it.”. If, for example, an overtrained player could get the approval from the physiotherapist to keep training, then it turns out that he could not train and needed to rest. The same could also happen with me as a physical trainer: if I go beyond the limit, then the player may be training too much, and if the player does not take care of himself (adequate rest, nutrition, etc), then nothing works” (PT 4)

Furthermore, S&CCs emphasize that everything that is done during planning is preventive and that not only individual or group work performed at low intensity on certain structures or musculature can be considered preventive work. Instead, there are numerous factors to consider, and focusing solely on the “preventive” ones would provide a very simplistic and limited perspective.

“Injury prevention is not something-specific; it includes everything we do. The pretense of finding specific exercises that focus on injury prevention is not correct. What prevents injuries and improves performance is a set of actions that we perform. We can implement many exercises, but if you do not sleep well, do not control the load, do not have minimum strength levels, etc., it does not matter what you do. And finally, training in court mimicking the specifics physical demands of competition is also highly preventive.”

(PT 6)

Considering the above, it is common for S&CCs to schedule these so-called “preventive” sessions, for both men’s and women’s teams and as a group (focusing on the main complementary structures that support sports performance and the general muscular and joint health of athletes), as well as individually (addressing each player’s deficits and weaknesses to minimize them).

“Even if we call it preventive, it is still good for me that we continue to train, for example, on ankle stability, ankle mobility, etc., even at low loads, regardless of the competitive demand” (PT 3), “maybe one day it’s more mobility and stretching and another day it’s more strength training with eccentrics exercise”

(PT 4)

The prescription of this “preventive” session varies considerably. The number of individual or group sessions scheduled, as well as their location, depends on each team’s weekly organization, player availability, or planned session type.

“Some players do this preventive training before court training and others in the gym on their own, at their preferable convenience” (PT 1), “players have a preventive-compensatory routine before each training session that they have to do independently” (PT 7), “players perform this preventive training on the court” (PT 8), and “every day before training the players perform stretching and mobility, although some players prefer to stay after training to do so. In addition, some players prefer to perform eccentric exercises after training because they believe it is better for them”

(PT 4)

#### 3.3.3. Measurements

Another essential aspect of S&CCs when monitoring players is the performance of tests that identify any player deficits. The main assessments include asymmetries tests, jump tests, ankle dorsiflexion evaluation, movement quality assessment, progressive load tests using encoders, and agonist-antagonist strength tests using dynamometers. These are planned for both male and female teams.

“First, we normally do different tests to assess how the player arrives” (PT 4), “We perform an initial assessment and then we follow up”

(PT 1)

### 3.4. Contextual and Relational Factors

This section compiles the results regarding various contextual factors and the relevance of the different multidirectional relationships that occur within a team environment throughout the season, all of which should be considered when optimizing training and achieving peak athletic performance.

#### 3.4.1. Contextual Factors

Unifying the participants’ views, several contextual factors were identified that should be considered by coaches and S&CCs when conducting training sessions, designing workouts, or planning programming. Key factors include the playing position, the moment within the season (such as schedule, competitive density, trips, and team ranking), the level of the opponent, the player’s context (age, body awareness, mood, previous sports education, and personal circumstances), or the results of the matches. It is important to note that these contextual factors are common to both male and female teams.

“I also give a lot of relevance to age, the experience level, and the current state of the player” (PT 3), “I try to get to know the players as much as possible so I can help them and guide them to the best of my ability. Also, when they are young, I try to help and educate them for their professional future” (PT 4), “when we start working with a new player, the first thing we do is to examine her previous history and evaluate her fitness, and from there, we begin to prescribe training.”

(PT 4)

“The season schedule is another key factor in establishing goals and allowing for an optimized periodization. However, initial planning may vary depending on the results during competition” (PT 7), “At certain times of the season, there is an increase in competitive density playing maybe five games in two weeks, which means playing every 3 or 4 days. In these cases, once this period of accumulation of games is over, I modify the type of training we do by changing, for example, the gym training or introducing more eccentric exercises that enable us to recover better. I also consider the most recent competitive weeks, the minutes played by athletes in competition, the moment of the season or of the amount of travelling” (PT 4), “the level of the opponent played on the weekend will also affect the weekly organization, as the players usually need more recovery time when playing against opponents in the upper part of the classification, which modifies the training sessions for that week.”

(PT 5)

“Each position has specific demands. For example, a pivot has to do more wrestling actions to fight and win the position, so those players need a greater orientation towards increasing strength levels (…), and a wing should train more speed-oriented, without, of course, doing a well-loaded basic strength. Therefore, we have to adapt the training to the different playing positions.”

(PT 6)

#### 3.4.2. Interpersonal Relationships

All participants, regardless of sex, agree that effective communication among team members is essential. These communications are multidirectional and include the relationships between the technical staff, which enable multidisciplinary work, and the relationships with the players, which are essential to achieving commitment, motivation, and confidence in their work.

“I find the relationship with the coach very relevant and, above all, being aware of his coaching style, his actions… At the beginning, as we start to work together, this is especially important” (PT 4), “it is essential to know the coach and his method of training” (PT 3), and “effective communication with the coach is also crucial. In the end, no matter how much we (physical trainers) know or do, if we do not know how to communicate with the coach and if he does not understand us, or does not pay attention to us, our work is useless.”

(PT 6)

“If you do a deadlift at 80% and then the coach wants to do transitions, then it is not well planned, no matter how good the transfer to real game may be”

(PT 4)

“The players are also very grateful for the feedback. If they realise that you make an effort, understand the training process, and see results, which in the end is the most important thing, they trust you greatly.”

(PT 4)

“Beyond the conditional aspect, one of the most important things is to have the players’ trust.” Being able to talk to them, both about sporting and personal issues, and to set goals will be fundamental to our work. If we do not have the confidence of the players, the first ones who have to change are us because we are not helping to improve the team.”

(PT 8)

Regarding interpersonal relationships, S&C coaches of women’s teams—each with prior experience in men’s teams—highlighted that the dynamics between technical staff and female players are notably more complex and challenging. They argued that differences in communication styles, team dynamics, and expectations contribute to a more complex relational landscape in women’s teams, which demands additional attention and tailored strategies to foster effective collaboration and performance.

“My communication style with female players differs notably from the approach I previously used with male players” (PT 3), “I engage in more extensive communication with the women, as I believe it helps them manage the diverse demands they face throughout the season” (PT 5), “I believe that with female players it is essential to provide individual explanations for decisions they may find challenging, while also offering encouragement to sustain their efforts.”

(PT 7)

### 3.5. Training Methods and Limitations

Finally, this section presents the participants’ perspectives on training methodologies and highlights the main limitations encountered in team management.

#### 3.5.1. Training Methodologies

Each S&CC applies different training methodologies based on their availability, resources, and contextual factors (playing position, opponent level, or season time). However, they emphasize the importance of prioritizing unilateral and dynamic training, implementing it progressively, using varied stimuli, intermittent actions with slight recovery and including recreational tasks that “trick” the athlete into achieving particular goals. The training methodologies employed by S&CCs are influenced more by the abovementioned factors than by the team’s gender.

“During resistance training in the gym, I start with bilateral exercises and progress to unilateral, (…). In the court, I consider that almost everything is unilateral due to the characteristics of the sport itself”

(PT 7)

“During strength training, I often use intermittent training, which intense effort with short rest, to guide it to the court, and circuit training, which enables me to better control the players”

(PT 8)

“I mainly perform neuromuscular, plyometric, and speed training. In addition, I try to orient the task according to the game model of the weekend’s opponent, if they play more with a fixed pivot or with four outsides”

(PT 5)

#### 3.5.2. Individualization

Individualization of training was undoubtedly the most frequently mentioned concept among all participants during the focus groups for both men’s and women’s teams, and they considered it essential for prescribing training programs. This individualization is transversal across all categories mentioned above, underscoring its fundamental importance and the need for both S&CCs and technical staff to integrate it into their planning.

“Individualization is crucial because each player has his own needs and his particular stage in the season”

(PT 8)

“First of all, we have to assess the player to identify his deficits and be able to individualize the strength training (…), and during the competitive weeks, I try to individualize the load of each player based on the different variables that we monitor (minutes of play, RPE, wellness…)”

(PT 3)

“Ultimately, many aspects depend on the player, on the individual preventive training they have, on when they think they should do it, on exercises that give them the feeling of being well, etc. Some players perform eccentric training on the same day of the match because they feel better on the court, so in the end, we should individualize many aspects based on each player.”

(PT 4)

#### 3.5.3. Limitations

S&CCs point out several key limitations: lack of time, particularly on the court, which often prevents them from providing adequate feedback, demonstrations, athletes’ instruction or conducting more appropriate warm-ups, suboptimal training facilities, lack of knowledge of new players when team changes occur, interference from uncontrollable variables during training, small technical teams with limited availability, restricted players availability and challenges in individualizing training to address each athlete’s specific needs. These limitations are widespread among S&CC teams in both men and women. However, participants noted that women’s futsal is less developed than men’s, and consequently, these limitations tend to be more pronounced.

“Due to lack of time, some training content cannot be done during the session and you have to find other times to do it, with the difficulties that this entails.” (PT 2), “I would like to have more time to warm up better and include compensatory and preventive content, but the reality is that I don’t have it.”

(PT 6)

“The players hardly ever do specific endurance work on the court with me. I have no time to train this with the players.”

(PT 1)

“I use RIR to prescribe strength training, but it is difficult for players to understand it and use it correctly.”

(PT 2)

“Many players do not know the correct exercise technique and I cannot always be present with them in the gym, which limits the exercise prescription and increases the injury risk of the players.”

(PT 1)

“Educating the athlete is essential, but the day-to-day logistics and the hurry due to the lack of time available make it very complicated (…) I wish I had more time to talk to the players.”

(PT 6)

“You must control many variables that are outside training”

(PT 8)

“Here, the training is over, and the physiotherapist is gone home in the first division. And then don’t call him in the afternoon because he has his practice”

(PT 4)

## 4. Discussion

To the authors’ knowledge, this is the first study that analyzes the usual practices of futsal S&CCs through focus groups. This approach enables us to know in an open and unrestricted manner the opinions of these professionals in the development of their professional practice. Although a previous study analyzed the futsal S&CCs usual practice methodologies [15], this study was conducted through a survey with closed-ended answers, which inherently limited the participant responses. The present study aimed to investigate contemporary practices among futsal S&CCs and to understand how they integrate their theoretical formation and prior experience into diverse, evolving professional contexts. The findings of this study offer an overview of current futsal S&CC practices and highlight key elements that they consider essential for fulfilling their roles. The main findings of the study were as follows: (a) neuromuscular and strength training two days per week throughout the season as the cornerstone of the players’ physical preparation and injury risk reduction; (b) meticulous monitoring of loads using different tools (e.g., GPS, LPS, RPE, Wellness assessments); (c) training individualization according to unique characteristics and contexts of each player and; (d) encouraging personal and professional relationships with the technical staff, which facilitated multidisciplinary collaboration in a productive and trust-based environment, as well as with the players, building mutual confidence and achieving greater implications and motivations.

Regarding the analysis of the performance model and competitive demands, S&CCs reported that strength, speed, and intermittent endurance are essential to futsal performance, a finding consistent with previous studies. Several studies have reported that lower limb strength plays a critical role in numerous game actions [49,50]. Enhanced lower limb strength increases motor unit recruitment, improves intra- and inter-muscular coordination, and boosts muscle mass, which is directly associated with better speed performance [30,51] and the execution of quick, explosive movements [52]. Meanwhile, other studies have indicated that movement speed is decisive in the game’s critical moments for executing high-intensity, short-duration actions [2,53,54,55]. Likewise, other authors have confirmed the relevance of aerobic training due to the nature of the sport [2,56] and anaerobic metabolism due to the high physical demands of professional futsal and the requirement of this metabolism in decisive actions of the game, such as counter-attack situations, retrieving the ball, or attempts to avoid a goal [56,57,58,59]. In addition, S&CCs emphasized the importance of repeating these high-intensity actions throughout the match, which agrees with other studies that concluded that the ability to repeat multiple high-intensity efforts is essential because of the high repetition demands of high-intensity actions present in futsal matches [7,60]. In this sense, it is noteworthy that S&CCs rely on evidence from the scientific literature to determine the training content included in their sessions.

Concerning load monitoring, S&CCs underscored the relevance of precise load control, which enables a detailed record of the cumulative load and recovery dynamics experienced by the players throughout the season. This view aligns with other studies that have confirmed that optimal load control is essential for ensuring proper athlete adaptation and effectively managing the balance between training and recovery to optimize sports performance [61,62]. Although several studies have confirmed the importance of monitoring load through the complementation of various internal and external load variables [62], several limitations hinder the implementation of these tools in current high-level futsal environments [63]. This is in contrast with what happens in other sports, such as soccer, where heart rate monitoring and GPS are common [64,65]. These differences between sports could be explained by the fact that GPS technology is not applicable in indoor environments [63]. Additionally, local positioning systems (LPS) require fixed installation in the team’s facilities, which poses practical challenges and renders them unsuitable for away matches. Moreover, LPS metrics are generally limited to accelerometry data, which restricts the analysis of distance and speed metrics [63]. The implementation of heart rate monitoring and GPS/accelerometry involves significant economic costs. Therefore, economic disparities between soccer and futsal may partly explain the limited use of these tools in futsal [15]. This, in turn, accounts for why S&CCs most commonly rely on RPE and wellness questionnaires for load monitoring, given their ease of use, implementation, and affordability. This approach is consistent with the findings of previous futsal studies [15] and other sports, such as rugby [66].

The injuries reported by the S&CCs were predominantly located in the lower body and were mainly muscular and ligamentous, which is consistent with findings from several previous studies [67,68,69,70]. Regarding the anatomical regions of these injuries, those affecting the ankle, knee, and thigh are predominant in both men and women, which is also in line with other studies [67,68,69,70]. Rui Pérez et al. [70] reported that the severity of injuries in futsal is usually slight (1–3 days) or mild (4–7 days), which enables most players to recover during the interval between games. This observation supports the perspective expressed by S&CCs in the focus groups. However, in contrast with other studies that reported most injuries in men’s [67] and women’s [68,71,72,73], futsal was moderate in severity (8–28 days). Among the multiple factors that could increase the risk of injury, S&CCs identified age as a particularly significant factor, which is in line with previous studies [72]. This underscores the need to consider age as a key factor in athlete preparation. Another important factor reported by S&CCs, which requires further research because of the lack of previous studies, is the type of court. According to the S&CCs, the playing surface appears to be associated with the occurrence of specific injuries, such as bone edemas or adductor muscle injuries. S&CCs have also reported a recent increase in anterior cruciate ligament (ACL) injuries, particularly in women’s futsal, a trend that could alter the current understanding of futsal injury profiles in the scientific literature. S&CCs should implement their preventive strategies in consideration of the main futsal injuries as well as other relevant factors, such as player age or the type of court on which the sport is usually practiced.

For neuromuscular and strength training, prioritizing unilateral over bilateral exercises is emphasized, given the sport’s predominantly one-leg demands (e.g., accelerations, changes of direction, shots). Although both training modalities enhance athletic performance [74,75], Stern et al. [76] highlighted the importance of unilateral specificity in soccer, and Gonzalo-Skok et al. [77] found that unilateral training yielded greater improvements in actions requiring unilateral force in basketball players. Ramirez-Campillo et al. [78] reported that unilateral training maximizes adaptations for game-specific, predominantly unilateral actions. Furthermore, studies by Gonzalo-Skok et al. [79,80] suggested that designing training based on the force vectors of specific movements is advantageous in team sports. The prescription of 2–3 days per week of these training sessions seems to be sufficient to achieve the sought adaptations. In addition, this training frequency was the most used by S&CCs, which is in line with that reported by other authors in several team sports [15,39].

S&CCs reported that their work is influenced by several contextual factors and relationships surrounding the context of the team in which they practice. Several studies have investigated the influence of playing position on player performance in futsal [35] and other sports, such as soccer [81], basketball [82], and rink hockey [83]. The incidence of schedule, ranking position, or match results has also been analyzed [35]. Several authors have also reported the importance of individual players’ physical (age, injury history, or level of experience) [72] and psychological characteristics in sports performance and injury prevention [84,85,86]. Personal relationships within a sports team environment are critical for optimizing all team members, both the technical staff and the players [14,87]. Moreover, López-Gajardo et al. [36] reported that this factor is even more relevant in women’s futsal than in men’s. Scientific literature reinforces the opinion expressed by S&CCs regarding the relevance of various factors that influence their professional practice. However, further research is needed to analyze the interpersonal relationships within teams and their relationship with the professional practices of all team members. This is a key aspect that S&CCs emphasized during the focus groups.

S&CCs report that they must adapt their training methodologies to the context they face, which is limited by suboptimal facilities, lack of time, players’ context, equipment, absence of advanced technology, or technical staff shortages. Spyrou et al. [15] reported that the primary concerns of futsal S&CCs were enhancing load monitoring capabilities (given the limitations of available technology), improving facilities, securing additional time, and expanding the technical staff. Weldon et al. [39] also found that limitations in technology, technical staff, facilities, equipment, and time were prevalent in other team sports, such as soccer or volleyball. The same results were found by Jones et al. [43] in rugby. In hockey, Ebben et al. [41] reported the need to improve training facilities and increase the influence over session design, underscoring the importance of strong personal and professional relationships within the technical staff. Therefore, S&CCs must adapt their practices to the particular conditions of each club.

Although analyzing the differences between male and female teams was not the primary objective of this study, the focus groups identified several key differences in the usual practices of S&CCs depending on the team’s sex. While the training methodologies remain similar, female teams often face unique challenges due to their specific demands, as documented by previous studies [8]. Men’s teams, although with their limitations as well, typically benefit from greater resources, which facilitate the adoption of advanced technologies (such as GPS), as evidenced in a recent systematic review [88]. In contrast, S&CCs working with women’s teams noted that athletes are generally less familiar with monitoring tools like wellness questionnaires, necessitating additional time for proper familiarization. In addition, consistent with previous research, the injury profiles differed between genders, with women’s teams exhibiting a higher prevalence of ACL injuries [30,51,53]. Moreover, in line with what was reported by Lopez-Gajardo et al. [36], interpersonal dynamics in women’s teams were described as more complex, requiring tailored communication strategies. These differences highlight the importance of implementing gender-specific adaptations in training, protocols, and injury prevention to optimize performance across both contexts.

The main limitation of this study is the small number of participants in the focus groups. Moreover, all participants belonged to Spanish league teams, which may overlook certain cultural aspects of S&CC practices from other geographical areas. Future research should replicate these focus groups with participants from different leagues and geographic contexts and consider concentrating on a specific aspect of S&CC practices to provide more in-depth insights. Additionally, an independent study of each gender, male and female, could allow for a more thorough examination of potential differences between the two.

## 5. Conclusions

The study revealed that elite futsal strength and conditioning coaches structure their work around five central areas: analyzing competitive demands, monitoring training loads, mitigating injury risks, considering contextual and interpersonal factors and implementing performance-oriented training methodologies. Coaches prioritize neuromuscular and strength training—conducted typically two days per week—as essential for enhancing lower-limb power and minimizing injuries. They rely heavily on tools such as RPE and wellness questionnaires for load monitoring because of practical constraints in using more advanced technologies. Although the practices implemented are largely effective, the coaches face persistent limitations, including limited time, inadequate facilities, scarce resources and challenges in individualizing training protocols. These constraints highlight the need for further optimization in both the planning and implementation of training programs. The insights provided by the focus groups offer practical applications for both current and aspiring S&CCs, supporting the development of more individualized, multidisciplinary, and context-specific training strategies in elite futsal environments. These conclusions contribute to a better understanding of current practices and challenges in elite futsal training and underscore avenues for future research aimed at enhancing athletic performance while reducing injury risk.

## Figures and Tables

**Table 1:** Participant characteristics.

Category	Classification	Number of Participants
Gender	Male	7
	Female	1
Age	<30	3
	30–35	4
	>35	1
Team category	First division	3
	Second division	5
Team gender	Male	4
	Female	4

**Table 2:** Overview of main results.

Categories	Subcategories	S&CC Current Practices and Key Considerations
Futsal performance model and competitive demands	Futsal performance	Lower-body strength training Intermittent training with incomplete and active recoveries to stress aerobic and anaerobic systems Speed training, especially acceleration
	Competitive demands	Playtime Player position-specific actions Distance covered
Load Monitoring	Load control and monitoring	Training load control Periodization: short- and long-term Questionnaire scores for wellness, RPE, TQR, and muscular quality New technologies (GPS)
Injuries	Main injuries	Ligament and muscle injuries Ankle, knee, hip, quadriceps, and adductor Women: higher prevalence of ACL and adductor injuries
	Strategies to minimize injury risk	Individual and group preventive sessions Strength training Synergy among coaches, S&CCs, medical services, and players Load control and periodization
	Measurements/Assessments	Individual deficit test Asymmetry, jumps, ankle dorsiflexion, movement quality assessment, progressive load, agonist–antagonist strength
Contextual and relational factors	Contextual factors	Playing position Season timing: schedule, trip, competitive density, and team ranking Opponent level Player context: age, body awareness, mood, previous sports education, and personal circumstances Match results
	Interpersonal relationships	Effective communication Technical staff communication: multidisciplinary work Player relationships: motivation and confidence
Training methods and limitations	Training methodologies	Based on availability, resources, and contextual factors Prioritize unilateral and dynamic training Intermittent actions with slight recovery
	Individualization	Essential for prescribing training programs Account for individual player characteristics
	Limitations	Lack of time Suboptimal training facilities Limited resources Insufficient staff Restricted player’ availability Individualization challenges

## Data Availability

The data that support the findings of this study are available from the corresponding author, upon reasonable request. The data are not publicly available due to privacy and ethical restrictions.
